# Clinicopathological factors associated with synchronous distant metastasis and prognosis of stage T1 colorectal cancer patients

**DOI:** 10.1038/s41598-021-87929-x

**Published:** 2021-04-22

**Authors:** Qiken Li, Gang Wang, Jun Luo, Bo Li, Weiping Chen

**Affiliations:** Department of Colorectal Surgery, Cancer Hospital of the University of Chinese Academy of Sciences (Zhejiang Cancer Hospital), 1 Banshan East Road, Hangzhou, 310022 Zhejiang China

**Keywords:** Cancer, Oncology, Risk factors

## Abstract

It is rare and understudied for patients with stage T1 colorectal cancer to have synchronous distant metastasis. This study was to determine the clinicopathological factors associated with distant metastasis and prognosis. T1 colorectal cancer patients diagnosed between 2010 and 2015 were obtained from the SEER database. Logistic regression was applied to determine risk factors related to distant metastasis. Cox-proportional hazard models were used to identify the prognostic factors for patients with distant metastasis. Among 21,321 patients identified, 359 (1.8%) had synchronous distant metastasis and 1807 (8.5%) had lymph node metastasis. Multivariate analysis revealed that younger age, positive serum CEA, larger tumor size, positive tumor deposit, perineural invasion, lymph node metastasis, histology of non-adenocarcinoma and poorer differentiation were significantly associated with the increased risk of synchronous distant metastasis. Older age, female, Black, positive CEA, positive lymph node metastasis, positive tumor deposit, larger tumor size, no chemotherapy, inadequate lymph node harvesting and no metastasectomy were correlated with worse survival in these patients with synchronous distant metastasis. Patients with metastasis to the liver displayed the highest rate of positive CEA. We conclude that T1 colorectal cancer patients with multiple risk factors need thorough examinations to exclude synchronous distant metastasis. Chemotherapy, adequate lymph node cleaning and metastasectomy are associated with improved survival for those patients with distant metastases. Positive serum CEA may be useful in predicting distant metastases in patients at stage T1.

## Introduction

Colorectal cancer is one of the most commonly diagnosed malignancies and the third leading cause of cancer-related deaths in the United States^1^. The JACC TNM staging system divides colorectal cancer into Tis and T1–T4 stages based on tumor invasion depth. Colorectal cancer limited to the inner layer or the submucosa of the bowel, is defined as the T1 stage. Most of the colorectal cancers at this stage are cured by surgical resection or even by endoscopic dissection. Only a small portion of these patients died due to recurrence and particularly distant metastasis of this disease. Distinguishing high-risk patients from most of the other low-risk patients is essential for providing appropriate therapy, improve survival and for avoiding unnecessary treatment.

Metastatic seeding occurs early in colorectal cancer^2^. It has been anecdotally reported that stage T1 colorectal cancer patients developed synchronous distant metastasis. A Japanese multicenter study previously reported only 0.2% (4 of 1806) synchronous liver metastasis in colorectal cancer patients at stage T1^3^. Another Japanese study found that 2 of 213 (0.9%) colorectal cancer patients at this stage developed synchronous liver metastasis^4^. A Japanese case study reported the synchronous liver metastasis without lymph node metastasis in a patient with T1 stage colorectal cancer (T1N0M1)^5^. A more recent case report identified a patient with a moderately differentiated T1 adenocarcinoma, had suspicious synchronous liver metastasis, which was not detected by preoperative imaging examination^6^. With the advent of detection technology, it is possible more T1colorectal patients are diagnosed with synchronous distant metastases. Among all patients at the T1 stage, this group of patients is expected to have the worst prognosis even after the most aggressive treatments. However, the clinicopathological features and prognostic factors of these patients have not been well studied.

This study was performed using the SEER national database to first compare clinicopathological features between stage T1 colorectal cancer patients with and without synchronous distant metastases. Logistic regression analyses were then applied to examine the risk factors correlated with distant metastases. Cox proportional hazard models were further employed to determine the risk factors associated with prognosis in patients with synchronous distant metastases. Findings from this population based study will assist to improve the management of stage T1 colorectal cancer patients.

## Results

### Baseline characteristics of patients with T1 colorectal cancer

Based on the exclusion and inclusion criteria, a total of 21,321 patients with stage T1 colorectal cancer were identified (Table 1). Among them, 63.3% were 60 years or older and 54.0% were male. Positive serum CEA was reported in 6.5% of patients. There were 78.0% colon cancers and 22.0% rectal cancers. Most (62.3%) of tumors had a size of 5 cm or smaller. Histologically, the majority of tumors (97.7%) were adenocarcinoma, and the rest were mucinous carcinoma (2.1%) or signet-ring cell carcinoma (0.2%). Most cancers were well (18.3%) or moderately (62.0%) differentiated. Lymph node metastases occurred in 8.5% (7.6% N1 and 0.9% N2) of patients. Tumor deposit was found in 0.8% of patients and perineural invasion was found in 0.9% of patients. Synchronous distant metastasis occurred in 379 (1.8%) patients. There were 8.4% of patients who received chemotherapy and 0.5% of patients who underwent metastasectomy (Table 1). The 5-year survival rate for stage T1 colorectal cancer in these patients was 82.8%.

**Table 1:** Characteristics of T1 stage colorectal cancer patients with or without distant metastasis.

	Overall	Distant metastasis		P value
	N = 21,321	No (M0) N = 20,942	Yes (M1) N = 379	
**Age (years)**				
< 60	7831 (36.7)	7666 (36.6)	165 (43.5)	0.0055
≥ 60	13,490 (63.3)	13,276 (63.4)	214 (56.5)	
**Gender**				
Male	11,508 (54.0)	11,298 (54.0)	210 (55.4)	0.5720
Female	9813 (46.0)	9644 (46.0)	169 (44.6)	
**Marital status**				
Married	12,054 (56.5)	11,867 (56.7)	187 (49.3)	< 0.0001
Single*	7364 (34.5)	7190 (34.3)	174 (45.9)	
Unknown	1903 (8.9)	1885 (9.0)	18 (4.8)	
**Race**				
White	16,350 (76.7)	16,069 (76.7)	281 (74.1)	0.0013
Black	2417 (11.3)	2355 (11.3)	62 (16.4)	
Other	2190 (10.3)	2154 (10.3)	36 (9.5)	
Unknown	364 (1.7)	364(1.7)	0 (0)	
**Tumor deposit**				
Negative	17,079 (80.1)	16,890 (80.7)	189 (49.9)	< 0.0001
Positive	176 (0.8)	157 (0.8)	19 (5.0)	
Unknown	4066 (19.1)	3895 (18.6)	171 (45.1)	
**Harvested lymph nodes**				
0–12	11,613 (54.5)	11,346 (54.2)	267 (70.5)	< 0.0001
> 12	9547 (44.8)	9440 (45.1)	107 (28.2)	
Unknown	161 (0.8)	156 (0.7)	5 (1.3)	
**Positive lymph nodes**				
No	12,798 (60.0)	12,712 (60.7)	86 (22.7)	< 0.0001
Yes	1691 (7.9)	1620 (7.7)	71 (18.7)	
Unknown	6832 (32.0)	6610 (31.6)	222 (58.6)	
**CEA status**				
Negative	7080 (33.2)	7015 (33.5)	65 (17.2)	< 0.0001
Positive	1376 (6.5)	1184 (5.7)	192 (50.7)	
Unknown	12,865 (60.3)	12,743 (60.9)	122 (32.2)	
**Liver metastasis**				
No	21,056 (98.8)	20,942 (100)	114 (30.1)	
Yes	265 (1.24)	0 (0)	265 (69.9)	
**Lung metastasis**				
No	21,243 (99.6)	20,942 (100)	301 (79.4)	
Yes	78 (0.4)	0	78 (20.6)	
**Insurance**				
No	374 (1.8)	361 (1.7)	13 (3.4)	0.0271
Yes	20,046 (94.0)	19,692 (94.0)	354 (93.4)	
Unknown	901 (4.2)	889 (4.3)	12 (3.2)	
**Localization**				
Colon	16,620 (78.0)	16,328 (78.0)	292 (77.0)	0.6676
Rectum	4701 (22.0)	4614 (22.0)	87 (33.0)	
**Histology**				
Adenocarcinoma	20,823 (97.7)	20,483 (97.8)	340 (89.7)	< 0.0001
Mucinous	450 (2.1)	418 (2.0)	32 (7.1)	
Signet-ring cell	48 (0.2)	41 (0.2)	7 (1.9)	
**Grade**				< 0.0001
Well differentiated	3895 (18.3)	3857 (18.4)	38 (10.0)	
Moderately differentiated	13,214 (62.0)	13,015 (62.2)	199 (52.5)	
Poorly differentiated	1182 (5.5)	1134 (5.4)	48 (12.7)	
Undifferentiated	173 (0.8)	169 (0.8)	4 (1.1)	
Unknown	2857 (13.4)	2767 (13.2)	90 (23.8)	
**Tumor size**				
≤ 5 cm	13,288 (62.3)	13,140 (62.7)	148 (39.1)	< 0.0001
> 5 cm	1122 (5.3)	1057 (5.1)	65 (17.2)	
Unknown	6911 (32.4)	6745 (32.2)	166 (43.8)	
**N stage**				
N0	19,094 (90.0)	18,861 (90.1)	233 (61.5)	< 0.0001
N1	1617 (7.6)	1531 (7.3)	86 (22.7)	
N2	190 (0.9)	157 (0.8)	33 (8.7)	
Unknown	420 (2.0)	393 (1.9)	27 (7.1)	
**Perineural invasion**				
No	16,865 (79.1)	16,665 (79.6)	200 (52.8)	< 0.0001
Yes	185 (0.9)	173 (0.8)	12 (3.2)	
Unknown	4271 (20.0)	4104 (19.6)	167 (44.1)	
**Radiotherapy**				
No	20,875 (97.9)	20,521 (98.0)	354 (93.4)	< 0.0001
Yes	446 (2.1)	421 (2.0)	25 (6.6)	
**Chemotherapy**				
No	19,527 (91.6)	19,416 (92.7)	111 (29.3)	< 0.0001
Yes	1794 (8.4)	1526 (7.3)	268 (70.7)	
**Metastasectomy**				
No	21,207 (99.5)	20,884 (99.7)	323 (85.2)	< 0.0001
Yes	114 (0.5)	58 (0.3)	56 (14.8)	
**Survival**				
No	981 (4.6)	762 (3.6)	219 (57.8)	< 0.0001
Yes	20,340 (95.4)	20,180 (96.4)	160 (42.2)	
**Overall survival**				
No	18,400 (86.3)	18,267 (87.2)	133 (35.1)	< 0.0001
Yes	2921 (13.7)	2675 (12.8)	246 (64.9)	
Median survival time (months)	40	41.0	19.0	
5-year survival rate (%)	82.8	95.9	33.2	< 0.0001

*Single includes divorces/separated/widowed/unmarried.

### Comparison of characteristics of patients with or without distant metastasis

The clinicopathological characteristics were compared in patients with or without synchronous distant metastasis (Table 1). Significantly more proportions of patients with synchronous distant metastasis were diagnosed at younger age, unmarried, Black, and had positive serum CEA, larger sized tumor (> 5 cm), non-adenocarcinoma, poorly or undifferentiated tumor, positive tumor deposit, perineural invasion and lymph node metastasis. In addition, significantly more proportions of patients with distant metastases received radiotherapy, chemotherapy and metastasectomy. During the median 40 months of follow-up, 762 (3.6%) patients without distant metastasis, and 219 (57.8%) patients with distant metastases died from the disease. The 5-year survival rates were 95.9% and 33.2% for patients without and with synchronous distant metastases respectively.

### Sites of distant metastases and their incidences in each year

The most common metastatic site was the liver (217 or 57.3%). Other included multiple-site metastases (49 or 12.9%), lung (33 or 8.7%), brain and bone (2 and 7, respectively, a total of 2.4%), and unknown distant sites (71 or 18.7%). Among multiple metastatic sites, there were 40 cases of liver and lung, 4 cases of liver and bone, 1 case of liver, lung and bone, and 4 cases of lung and bone (Supplemental data Table S1). The number of patients with distant metastases was calculated based on diagnosis years (Supplemental data Fig. S1).

### Clinicopathological factors predicting distant metastasis

**Figure 1:**
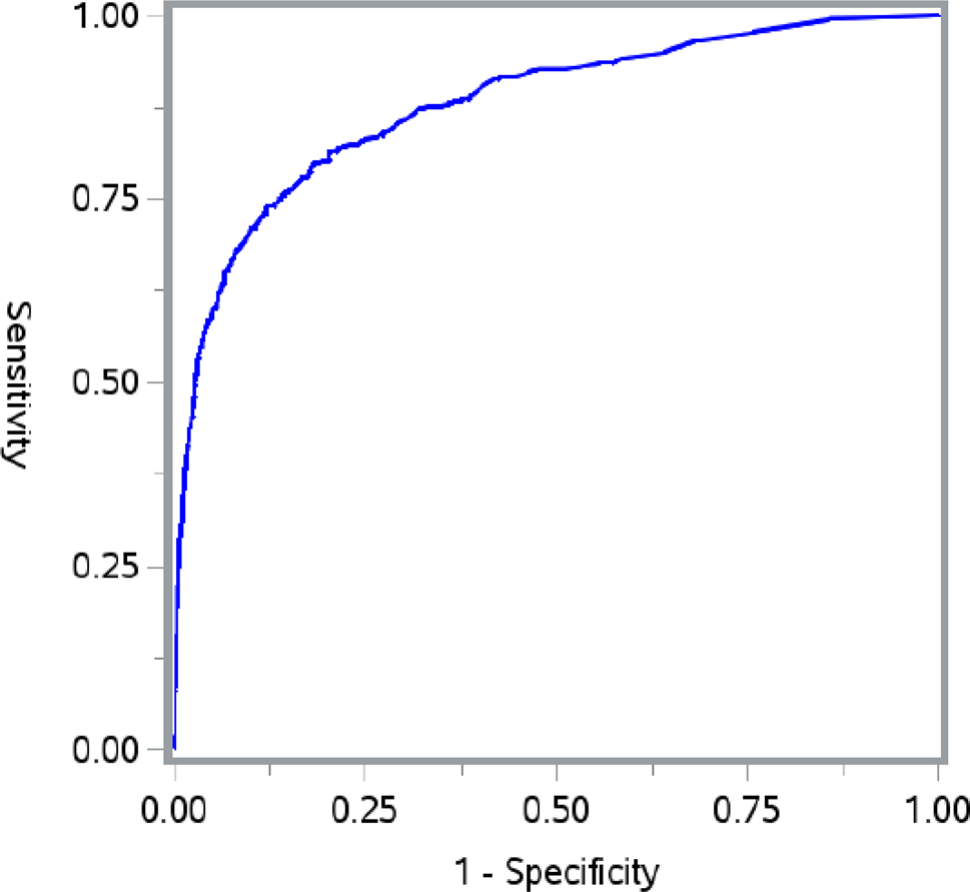
Receiver operating characteristics (ROC) curves for the model in differentiating stage T1 colorectal cancer with distant metastases from those without distant metastases. The area under the ROC curve was 0.879.

Logistic regression analysis was applied to determine the clinicopathological factors correlated with the risk of synchronous distant metastasis. Both univariate and multivariate analyses showed that younger age, positive serum CEA, larger tumor size (> 5 cm), non-adenocarcinoma, higher grade, positive tumor deposit, perineural invasion and lymph node metastasis were significantly associated with an increased risk of distant metastasis (Table 2). A receiver operating characteristic (ROC) curve was constructed to evaluate the performance of the predicting model built based on these risk factors (Fig. 1). The area under the ROC curve was 0.879 in distinguishing stage T1 colorectal cancer patients with synchronous distant metastases, from those without distant metastases.

**Table 2:** Risk factors associated with the distant metastasis in patients with T1 stage colorectal cancer.

	Univariate		Multivariate	
	OR (95% CI)	P value	OR (95% CI)	P value
**Age(years)**				
< 60	1		1	
≥ 60	0.7 (0.6–0.9)	0.0057	0.7 (0.6–0.9)	0.0104
**Gender**				
Male	1			
Female	0.9 (0.8–1.2)	0.5720		
**Marital status**				
Married	1			
Single*	1.5 (1.2–1.9)	< 0.0001		
Unknown	0.6 (0.4–0.98)	0.0435		
**Race**				
White	1			
Black	1.5 (1.1–2.0)	0.0040		
Other	1.0 (0.6–1.4)	0.7998		
Unknown	1.0 (0.7–1.4)	0.7480		
**CEA**				
Negative	1		1	
Positive	17.5 (13.1–23.3)	< 0.0001	14.9 (11.0–20.2)	< 0.0001
Unknown	1.0 (0.8–1.4)	0.8325	0.8 (0.6–1.1)	0.0975
**Tumor localization**				
Colon	1			
Rectum	1.1 (0.8–1.3)	0.6676		
**Tumor size**				
≤ 5 cm	1		1	
> 5 cm	5.5 (4.1–7.4)	< 0.0001	4.0 (2.8–5.7)	< 0.0001
Unknown	2.2 (1.7–2.7)	< 0.0001	1.8 (1.4–2.4)	< 0.0001
**Histology**				
Adenocarcinoma	1		1	
Mucinous	4.6 (3.2–6.7)	< 0.0001	4.1 (2.6–6.4)	< 0.0001
Signet-ring cell	10.3 (4.6–23.1)	< 0.0001	5.9 (2.2–16.0)	0.0004
**Grade**				
Well/moderately differentiated	1			
Poorly/un-differentiated	2.8 (2.1–3.9)	< 0.0001	1.9 (1.3–2.78)	0.0009
Unknown	2.3 (1.8–3.0)	< 0.0001	1.5 (1.2–2.0)	0.0028
**Tumor deposit**				
Negative	1		1	
Positive	10.8 (6.6–17.8)	< 0.0001	2.5 (1.3–4.8)	0.0041
Unknown	3.9 (3.2–4.8)	< 0.0001	3.1 (2.4–4.1)	< 0.0001
**Perineural invasion**				
No	1		1	
Yes	5.8 (3.2–10.5)	< 0.0001	2.5 (1.2–5.3)	0.0156
Unknown	3.4 (2.8–4.2)	< 0.0001	2.1 (1.6–2.7)	< 0.0001
**Lymph**				
0–12	1			
> 12	0.5 (0.4–0.6)	< 0.0001		
Unknown	1.4 (0.6–3.3)	0.5003		
**Positive lymph node**				
No	1			
Yes	6.5 (4.7–8.9)	< 0.0001		
Unknown	4.9 (3.9–6.4)	< 0.0001		
**N stage**				
N0	1		1	
N1	4.6 (3.5–5.9)	< 0.0001	4.0 (3.0–5.4)	< 0.0001
N2	17.0 (11.4–25.3)	< 0.0001	8.5 (5.0–14.2)	< 0.0001
Unknown	5.6 (3.7–8.4)	< 0.0001	3.0 (1.9–4.8)	< 0.0001
**Insurance**				
Non insured	1			
Insured	0.5 (0.3–0.9)	0.0155		
Unknown	0.4 (0.2–0.8)	0.0154		

*CI* confidence interval, *OR* odds ratio. *Single includes divorced/separated/widowed/unmarried. The formula generated by the multivariate model predicts OR = e^(− 5.81 − 0.30 × age (≥ 60) + 1.36 × tumor size (> 5 cm) + 0.53 × tumor size (unknown) + 2.70 × CEA (positive) –  0.27 × CEA (unknown) + 1.42 × mucinous adenocarcinoma + 1.78 × Signet ring cell carcinoma + 0.98 × tumor deposit (positive) + 1.13 × tumor deposit (unknown) + 0.62 × poorly or un-differentiated + 0.43 × unknown differentiation + 0.92 × perineural invasion (positive) + 0.74 × perineural invasion (unknown) + 1.39 × N stage (N1) + 2.14 × N stage (N2) + 1.11 × unknown N stage).

### Prognostic factors for stage T1 colorectal patients with synchronous distant metastasis

**Figure 2:**
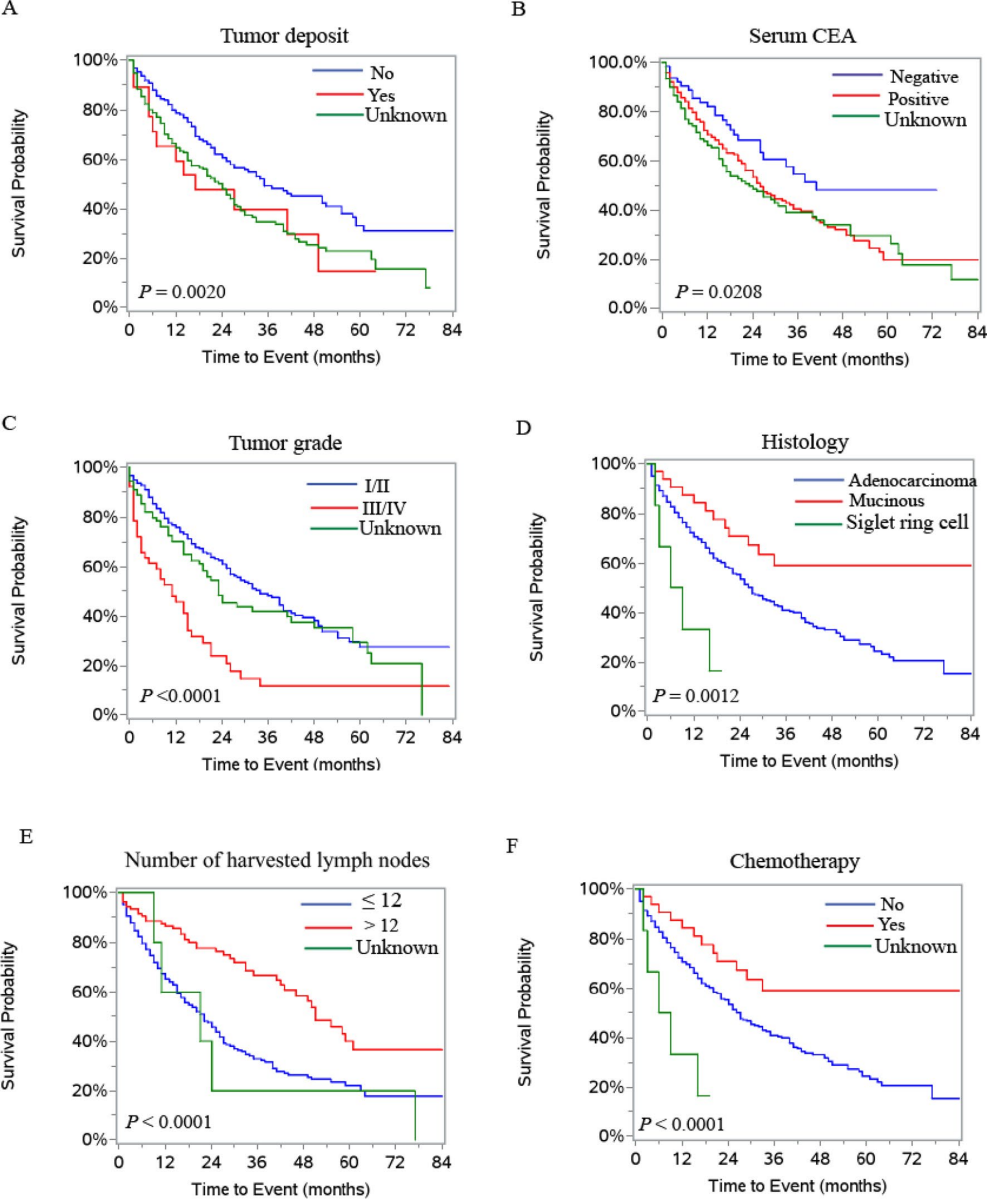
Kaplan–Meier survival curves for patients with stage T1 colorectal cancer. (**A**) Tumor deposits. (**B**) Serum CEA. (**C**) Tumor grade. (**D**) Histology. (**E**) Number of harvested lymph nodes. (**F**) Chemotherapy.

Cox-proportional hazard regression models were applied to analyze risk factors associated with survival in patients with distant metastases. Both univariate and multivariate results showed that age, gender, race, CEA status, tumor size, histology, grade, tumor deposit, number of lymph node harvested, lymph node metastasis, metastatic sites, chemotherapy and metastasectomy were significantly associated with cancer specific survival (Table 3). The survival curves of the risk factors are presented in Fig. 2A–F and Supplemental Fig. S2A–S2F).

**Table 3:** Prognostic factors for cancer specific survival and overall survival for patients with stage T1 colorectal cancer.

	Cancer specific survival				Overall survival			
	Univariate		Multivariate		Univariate		Multivariate	
	HR (95% CI)	P value	HR (95% CI)	P value	HR (95% CI)	P value	HR (95% CI)	P value
**Age**								
< 60	1		1		1		1	
≥ 60	1.7 (1.3–2.2)	0.0002	1.7 (1.3–2.3)	0.0004	1.7 (1.3–2.2)	< 0.0001	1.7 (1.3–2.2)	0.0002
**Gender**								
Male	1		1		1			
Female	0.9 (0.8–0.998)	0.0456	1.4 (1.02–1.8)	0.0373	0.9 (0.9–1.0)	0.0773		
**Marital status**								
Married	1				1			
Single*	1.5 (1.1–2.0)	0.0036			1.5 (1.2–2.0)	0.0010		
Unknown	0.8 (0.4–1.6)	0.5992			0.9 (0.5–1.7)	0.7873		
**Race**								
White	1		1		1		1	
Black	1.5 (1.1–2.1)	0.0203	1.7 (1.2–2.4)	0.0054	1.5 (1.03–1.9)	0.0319	1.6 (1.2–2.3)	0.0045
Other	0.7 (0.4–1.2)	0.1633	0.8 (0.5–1.5)	0.5450	0.6 (0.4–1.1)	0.0918	0.8 (0.5–1.3)	0.3107
**CEA**								
Negative	1		1		1			
Positive	1.7 (1.1–2.7)	0.0132	1.9 (1.2–3.0)	0.0062	1.4 (0.97–2.1)	0.0726		
Unknown	1.9 (1.2–2.9)	0.0075	1.7 (1.1–2.8)	0.0250	1.5 (1.03–2.3)	0.0339		
**Tumor localization**								
Colon	1				1			
Rectum	1.4 (1.04–1.9)	0.0254			1.4 (1.02–1.8)	0.0312		
**Tumor size**								
≤ 5 cm	1		1		1			
> 5 cm	1.5 (1.01–2.2)	0.0424	1.6 (1.05–2.4)	0.0300	1.3 (0.9–1.9)	0.1153		
Unknown	1.7 (1.3–2.3)	0.0004	1.6 (1.1–2.2)	0.0074	1.6 (1.2–2.1)	0.0020		
**Histology**								
Adenocarcinoma	1		1		1		1	
Mucinous	0.5 (0.3–0.8)	0.0093	0.5 (0.2–0.9)	0.0255	0.4 (0.2–0.6)	0.0001	0.5 (0.3–0.9)	0.0028
Signet-ring cell	3.2 (1.3–7.8)	0.0115	1.4 (0.5–3.9)	0.5235	3.2 (1.4–7.3)	0.0047	1.6 (0.6–3.9)	0.3342
**Grade**								
Highly/moderately differentiated	1		1		1		1	1
Poorly/un-differentiated	2.6 (18–3.7)	< 0.0001	2.5 (1.7–3.8)	< 0.0001	2.5 (1.8–3.6)	< 0.0001	2.3 (1.6–3.4)	< 0.0001
Unknown	1.2 (0.9–1.7)	0.1942	0.9 (0.6–1.3)	0.5723	1.2 (0.9–1.7)	0.1522	1.0 (0.8–1.4)	0.7854
**Tumor deposit**								
Negative	1		1		1		1	
Positive	1.7 (0.9–3.1)	0.0812	3.0 (1.5–5.7)	0.0011	1.7 (0.96–2.9)	0.0667	2.4 (1.3–4.4)	0.0038
Unknown	1.2 (1.2–2.1)	0.0007	1.1 (0.8–1.5)	0.6641	1.4 (1.1–1.9)	0.0053	1.0 (0.8 -1.3)	0.9698
**Perineural invasion**								
No	1				1			
Yes	0.5 (0.2–1.5)	0.2203			0.7 (0.3–1.6)	0.4026		
Unknown	1.0 (0.8–1.3)	0.8293			1.0 (0.7–1.2)	0.7874		
**Harvested lymph nodes**								
0–12	1		1		1		1	
> 12	0.4 (0.3–0.6)	< 0.0001	0.5 (0.3–0.7)	0.0005	0.4 (0.3–0.6)	< 0.0001	0.4 (0.3–0.6)	< 0.0001
Unknown	1.2 (0.5 -3.0)	0.6387	1.1 (0.3 -3.1)	0.5362	1.1 (0.5–2.7)	0.8302	0.9 (0.4–2.4)	0.8520
**Positive lymph nodes**								
No	1				1			
Yes	2.1 (1.3–3.5)	0.0031			2.0 (1.3–3.2)	0.0032		
Unknown	3.3 (2.2–5.0)	< 0.0001			3.0 (2.1–4.5)	< 0.0001		
**N stage**								
N0	1		1		1		1	
N1	0.9 (0.7–1.3)	0.1905	1.0 (0.7–1.4)	0.8494	0.9 (0.7–1.2)	0.4975	0.9 (0.6–1.2)	0.4264
N2	1.6 (1.05–2.6)	0.0302	1.9 (1.1–3.3)	0.0160	1.5 (1.01–2.4)	0.0463	1.9 (1.2–3.2)	0.0096
Unknown	2.1 (1.4–3.4)	0.0012	1.7 (1.1–2.8)	0.0294	1.9 (1.2–3.0)	0.0042	1.5 (0.9–2.4)	0.1041
**Metastasis**								
Multiple sites	1		1		1		1	
liver	0.5 (0.3–0.7)	0.0002	0.5 (0.4–0.8)	0.0017	0.5 (0.4–0.7)	0.0001	0.6 (0.4–0.8)	0.0043
lung	0.4 (0.2–0.7)	0.0025	0.2 (0.1–0.4)	< 0.0001	0.4 (0.3–0.8)	0.0030	0.2 (0.1–0.4)	< 0.0001
Brain or bone	1.7 (0.7–4.0)	0.2495	1.2 (0.5–3.2)	0.6648	2.0 (0.9–4.2)	0.0831	1.2 (0.5–2.9)	0.6100
other	0.3 (0.2–0.6)	< 0.0001	0.3 (0.2–0.5)	< 0.0001	0.4 (0.2–0.6)	< 0.0001	0.4 (0.2–0.6)	0.0001
**Chemotherapy**								
No	1		1		1		1	
Yes	0.5 (0.4–0.7)	< 0.0001	0.4 (0.3–0.6)	< 0.0001	0.5 (0.4–0.6)	< 0.0001	0.4 (0.3–0.5)	< 0.0001
**Radiotherapy**								
No	1				1			
Yes	1.3 (0.8–2.1)	0.2303			1.2 (0.7–1.8)	0.5068		
**Metastasectomy**								
No	1		1		1		1	
Yes	0.4 (0.2–0.6)	< 0.0001	0.5 (0.3–0.7)	0.0002	0.3 (0.2–0.5)	< 0.0001	0.4 (0.2–0.6)	0.0002

*HR* hazard ratio, *CI* confidence interval; *Single includes divorces/separated/widowed/unmarried.

Univariate and multivariate survival analysis revealed that age, race, histology, tumor grade, tumor deposit, harvested lymph node number, lymph node metastases, metastatic sites, chemotherapy and metastasectomy were associated with overall survival in stage T1 colorectal patients with synchronous distant metastasis (Table 3).

### Tumor histology and survival

It was surprising that mucinous adenocarcinoma was associated with improved cancer specific survival and overall survival in stage T1 colorectal cancer patients with synchronous distant metastases (Table 3 and Fig. 2D). We therefore analyzed the correlation between tumor histology and survival in all stage T1 patients and patients without synchronous distant metastases. The results indicated that adenocarcinoma was associated with better cancer specific and overall survival in all stage T1 patients or patients without synchronous distant metastases (Supplemental data Fig. S3A–S3D).

### Serum CEA status and metastatic sites

Stage T1 colorectal cancer patients without distant metastases had a relatively low rate (5.7%) of positive serum CEA. The rate of positive serum CEA was significantly increased to approximately 30% in patients with synchronous non-liver distant metastases, and further significantly increased to 60% in patients with synchronous distant metastases to liver (Table 4).

**Table 4:** Correlation between serum CEA and distant metastasis in patients with T1 colorectal cancer.

	Status of serum CEA n (%)		
	Negative (n = 7080)	Positive (n = 1376)	Unknown (n = 12,865)
**Distant metastasis**			
Multiple sites	7 (14.3)	27 (55.1)	15 (30.6)
Liver only	28 (12.9)	132 (60.8)	57 (26.3)
Lung only	10 (30.3)	9 (33.3)	14(42.2)
Brain or bone	2 (22.2)	3 (33.3)	4 (44.4)
Other organs	18 (25.4)	21 (29.6)	32 (45.1)
No distant metastasis	7015 (33.5)	1184 (5.7)	12,743 (60.8)

## Discussion

T1 colorectal cancer patients with synchronous distant metastases have a poor prognosis even after the most aggressive treatment. Due to the very low incidence, their clinicopathological characteristics have not been well characterized. Using the SEER database, this population-based study identified multiple clinicopathological factors associated with the increased risk of synchronous distant metastases: diagnosis at a younger age, positive serum CEA, larger tumor size, positive tumor deposit, perineural invasion and lymph node metastasis, histology of non-adenocarcinoma and poorer differentiation. The predicting model using these risk factors had an area under the ROC of 0.879 in distinguishing patients with distant metastasis from those without distant metastasis. Most described risk factors have been associated with increased risk of recurrence due to heightened aggressive metastases in advanced staged colorectal cancer; however, very few have been previously reported to be risk factors related to lymph node metastases and recurrence in T1 colorectal cancer patients^5,7–9^. It is noted that a previous study revealed that 4 (2.1%) of 195 consecutive stage T1 colorectal cancer patients had synchronous distant metastasis^7^, a comparable incidence rate as ours.

This study indicated that patients with T1 colorectal cancer who have all or most of the risk factors are very likely to have distant metastases. Through combination with molecular markers and advanced screening technology, these risk factors may be useful to distinguish T1 colorectal cancer patients with high risk of distant metastasis, from those with low-risk of distant metastases.

CEA is a valuable serum biomarker in differential diagnosis, disease monitoring and evaluation of therapeutic efficacy in colorectal cancer. This study showed that patients with positive CEA had a significantly worse survival and a higher risk of distant metastasis. Moreover, T1 colorectal cancer patients without metastasis had a low percentage of had positive serum CEA, which was significantly increased in patients with non-liver distant metastases, and further significantly increased in patients with liver metastases. These results suggest that serum CEA is particularly useful to screen synchronous liver metastasis in patients with T1 colorectal cancer. A previous study reported that CEA was as effective as computed tomography (CT) imaging in assessing the response of chemotherapy in colorectal cancer patients with liver metastases^10^. CEA has been shown to play a direct role in assisting metastasis of colorectal cancer cells to the liver and therefore it is a potential target in the treatment of liver metastasis^11^.

The AJCC edition 7-TNM staging defines tumor deposits as a macroscopic or microscopic tumor nest or nodule in adjacent adipose tissue, without lymph node structure^12^. However, tumor deposits are often overlooked in TNM staging, particularly when they coexist with lymph node metastasis. Many clinicians are confused about the actual prognostic impact of tumor deposits. This study indicated that less than 1% of T1 colorectal cancer patients had tumor deposits. Multivariate Cox proportional hazard analysis showed that tumor deposits were significantly associated with an increased risk of distant metastasis, and adverse survival outcomes. Multiple studies and reviews have reported that tumor deposits are associated with increased local recurrence and distant metastasis rates, and overall survival in colorectal cancer patients^13–15^. Due to the limit of the database, it is not possible to determine other characteristics of tumor deposits (such as their location, diameter, and shape) and their correlation with prognosis.

Primary colorectal mucinous adenocarcinoma and signet-ring cell carcinoma are two rare subtypes of colorectal cancer with a poorer prognosis than typical adenocarcinoma^16,17^. Mucinous carcinoma may respond poorly to chemotherapy than adenocarcinoma, as mucins may be an obstacle to drug delivery. This study revealed that mucinous adenocarcinoma was associated with worse survival in all T1 patients or patients without synchronous distant metastases. However, it was associated with improved cancer survival in T1 colorectal cancer patients with synchronous distant metastases. The underlying mechanism is still unknown. This finding needs to be corroborated in future studies. The association between mucinous adenocarcinoma and improved prognosis is inconsistent with results in colorectal cancer patients at other stages, as reported previously^16,18–22^.

Perineural invasion is a pathological process in which the tumor infiltrates the nerve structure and spreads along the nerve sheath^23^. The perineural invasion has been served as an important risk factor related to recurrence and a low survival rate in colorectal cancers and other cancers^24–26^. Perineural invasion is also being used as a novel target to block tumor progression and improve survival^23^. This study found a very low rate (0.9%) of perineural invasion in T1 colorectal cancer patients. It was significantly correlated with the increased risk of synchronous distant metastases, but not associated with the survival in these T1 colorectal cancer patients with synchronous distant metastases.

National Comprehensive Cancer Network (NCCN) guidelines recommend sampling at least 12 lymph nodes for adequate staging of colorectal cancer^27^. Adequate lymph node cleaning has been associated with better prognosis in colorectal cancer patients at other stages^28,29^, but not in others^30,31^. This study revealed that harvesting over 12 lymph nodes was associated with increased survival in stage T1 colorectal cancer patients with distant metastasis, which supports harvesting an adequate number of lymph nodes to improve the survival in these high-risk stage T1 patients.

Stage T1 colorectal cancer patients with synchronous distant metastasis are treated the same as patients with distant metastasis at other stages^32^. In the past decade, the efficacy of systematic chemotherapy, particularly with the recently developed personalized target therapy, has dramatically improved in the treatment of patients with metastatic colorectal cancer. However, the benefit of chemotherapy has not been previously studied in T1 patients with synchronous distant metastasis. In this study, 70.3% of patients with synchronous distant metastasis received chemotherapy, whereas only 7.3% of patients without distant metastasis received chemotherapy. Chemotherapy was associated with a significantly improved survival in stage T1 colorectal cancer patients with distant metastasis. Similar to this finding, a previous study using the National Cancer Database reported that adjuvant chemotherapy significantly improved survival in stage T1 colorectal patients with lymph node metastasis^33^. The survival benefit of chemotherapy supports its use for these high-risk T1 colorectal cancer patients.

Distant metastases are the main cause of death in colorectal cancer patients. Both metastatic burden and its involvement of vital organs are important prognostic factors for metastatic colorectal cancer^34^. This study indicated that patients with metastases to multiple-sites, or bone, or brain had significantly worst survival compared to those with liver or lung metastasis alone. Among all patients with synchronous distant metastasis, 56 (14.8%) patients received metastasectomy. Multivariate survival analyses using Cox proportional hazards models revealed that metastasectomy was significantly associated with increased survival in stage T1 colorectal cancer patients with synchronous distant metastasis. The metastasectomy in metastatic colorectal cancer demonstrated the survival beneficial in previous studies^34–37^. Simultaneous colorectal and minor hepatic resections are considered to be safe for most patients with synchronous liver metastases^38^.

This population-based study has several limitations. As a retrospective study, there is an inherent bias in patient selection. Some important clinical information possibly related to prognosis is not included in the database. There is no data regarding the depth of invasion to the submucous membrane, the edge of the surgical margin. The size and number of metastases at each site are unknown. It is unclear if patients received neoadjuvant, or adjuvant chemotherapy, or both; Their detailed chemotherapy regimen, dose and duration were also unclear. It is unknown whether metastasectomy was performed before, at the same time with, or after surgical resection of colorectal cancer. The database lacks information on complications, recurrence and the metachronous metastasis after the treatments as well. Due to lack of detailed information, only 21,321 (51.6%) of 41,312 T1-stage patients were selected in this study. The strength of this study is that it is the first time data has been reported with a large number of T1 colorectal patients having synchronous distant metastasis.

## Conclusions

Younger age, positive serum CEA, larger tumor size, positive tumor deposit, perineural invasion and lymph node metastasis, histology of non-adenocarcinoma and poorer differentiation, are associated with increased risk of distant metastasis, in T1 colorectal cancer patients. Thorough examinations to exclude distant metastasis are needed for T1 colorectal cancer patients with multiple risk factors. Chemotherapy, adequate lymph node cleaning and metastasectomy are associated with improved survival for those patients with distant metastases. Positive serum CEA may be useful in predicting distant metastases, particularly liver metastases.

## Patients and methods

**Figure 3:**
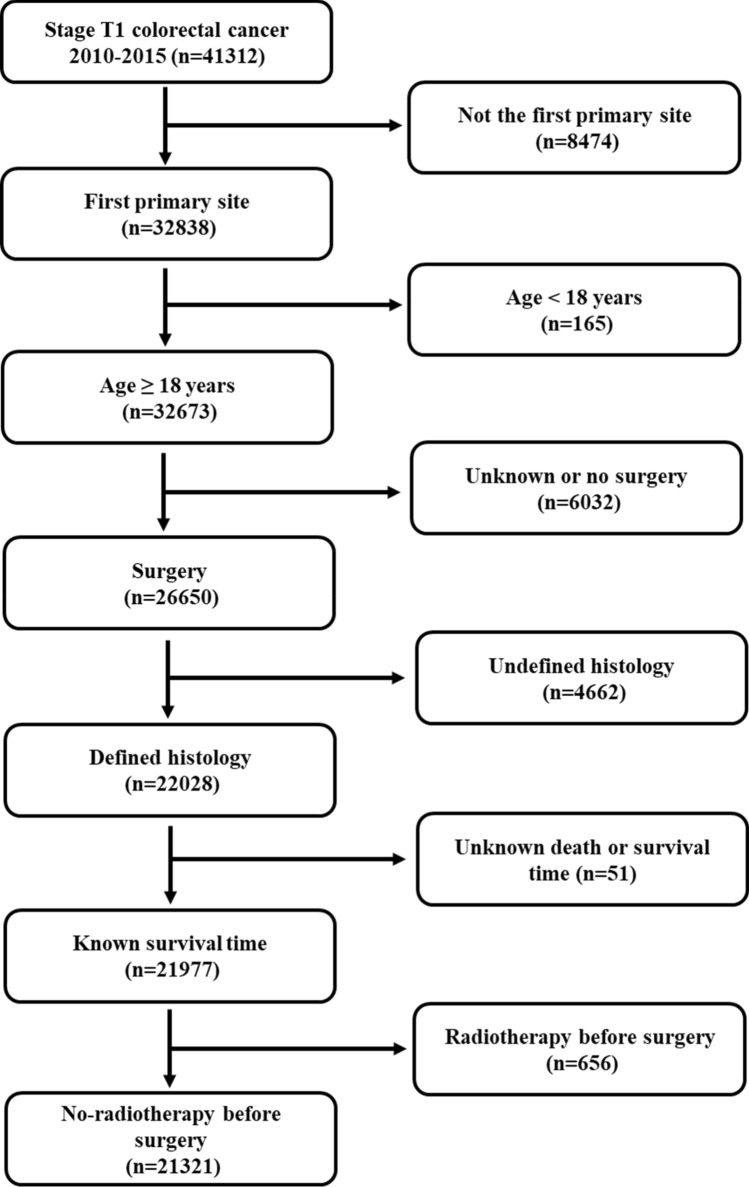
Flowchart of data selection based on inclusion and exclusion criteria.

The study cohort was obtained from the SEER database using SEER*Stat 8.3.6. According to the Site Recode Classifications, patients with colon (C18.0–18.9, and C26.0) and rectal (C19.9 and C20.9) cancers were identified from the SEER database. The inclusion criteria used for this study were: (a) the first primary tumor; (b) age of 18 or older; (c) diagnosed between 2010 and 2015; (d) surgery was performed with pathological confirmation; (e) pathological stage T1 according to AJCC 7th edition. The exclusion criteria were: (a) patients received preoperative radiation (downstage T stage); (b) histology other than adenocarcinoma, mucinous adenocarcinoma and signet ring cell carcinoma; (c) patients with unknown survival status or time (Fig. 3).

The following variables were gathered: age at diagnosis, gender, marital status, race, the status of serum CEA, tumor size, histologic type, differentiation, the status of lymph node metastasis, number of lymph nodes harvested, metastatic sites, cancer specific survival and overall survival months. The histological types were categorized using the ICD-O-3 (International Classification of Disease for Oncology, 3rd edition) coding schema: conventional adenocarcinoma (8010, 8020- 8022, 8140–8141, 8144–8145, 8210–8211, 8220–8221, 8230–8231, 8260–8263), mucinous adenocarcinoma (8470, 8472–8473, 8480–8481); and signet-ring cell carcinoma (8490)^39^. The tumor grades were grouped as: Grade I (well differentiated); Grade II (moderately differentiated); Grade III (poorly differentiated); Grade IV (undifferentiated or anaplastic lesions). The metastasectomy was defined by “non-primary surgical procedure to a distant site” in the record of “surgery other regional/distant (2003 +)”^40^. The survival time was calculated from the month of diagnosis to death or censored. The last follow-up month in the database was December of 2016.

This study was based on public data from the SEER database without interacting with human subjects or using personal identifying information. This research was therefore exempted from review by the Human Subjects Committee of Institutional Review Board of our hospital.

### Statistical analysis

The frequency and percentage were calculated for clinicopathological variables and a Chi-square test was used to compare the patients with and without distant metastasis. Kaplan–Meier curves were generated for cancer specific and overall survival and the significance was assessed using a log-rank test. Cox proportional hazards models were used in the univariate and multivariate analyses. Two-sided p values < 0.05 were considered statistically significant. The SAS software (Version 9.3, SAS Institute Inc., Cary, NC, USA) was used to analyze data.

### Ethics approval and consent to participate

As the SEER dataset is publicly available and de-identified, therefore, the ethical approval was waived by the ethics committee of our hospital.

## Supplementary Information

Supplementary Information.

## Data Availability

The datasets used in this manuscript are available in the Surveillance, Epidemiology, and End Results (SEER) database (http://www.seer.cancer.gov) of the National Cancer Institute.
