# Supplementary material for: Clinicopathological factors associated with synchronous distant metastasis and prognosis of stage T1 colorectal cancer patients
Source: Sci Rep. 2021 Apr 22;11:8722. doi: 10.1038/s41598-021-87929-x (PMC8062534; doi:10.1038/s41598-021-87929-x)

Clinicopathological factors associated with synchronous distant metastasis and prognosis of stage T1 colorectal cancer patients

Qiken Li^1^, Gang Wang^1^, Jun Luo^1^, Bo Li^1^, Weiping Chen^1,*^

^1^ Department of Colorectal Surgery, Cancer Hospital of the University of Chinese Academy of Sciences (Zhejiang Cancer Hospital), Hangzhou, Zhejiang 310022, China.

^*^Corresponding author:

Weiping Chen, MD

Department of Colorectal Surgery

Cancer Hospital of the University of Chinese Academy of Sciences (Zhejiang Cancer Hospital)

1 Banshan East Road

Hangzhou, Zhejiang 310022

China

Tel: 86-571-88128201

E-mail: [cwp819@yahoo.com](mailto:cwp819@yahoo.com)

Supplemental data

Table S1. Metastatic sites in patient with T1 colorectal cancer

| **Metastatic site** | **N (%)** |
| --- | --- |
| **Liver** | 217 (57.3) |
| **Multiple sites (overall)** | 49 (12.9) |
| **Liver and lung** | 40 (10.6) |
| **Liver and bone** | 4 (1.1) |
| **Lung and bone** | 1 (0.3) |
| **Liver, lung and bone** | 4 (1.1) |
| **Lung alone** | 33 (8.7) |
| **Bone alone** | 7 (1.9) |
| **Brain alone** | 2 (0.5) |
| **Other organs** | 71 (18.7) |

Fig. S1. Percentage of T1 colorectal cancer patients with distant metastases from 2010-2015

Fig. S2. Kaplan-Meier survival curves for patients with stage T1 colorectal cancer.

(A) Age at diagnosis. (B) Gender. (C) Race. (D) Tumor size. (E) Metastatic sites. (F) Metastasectomy.


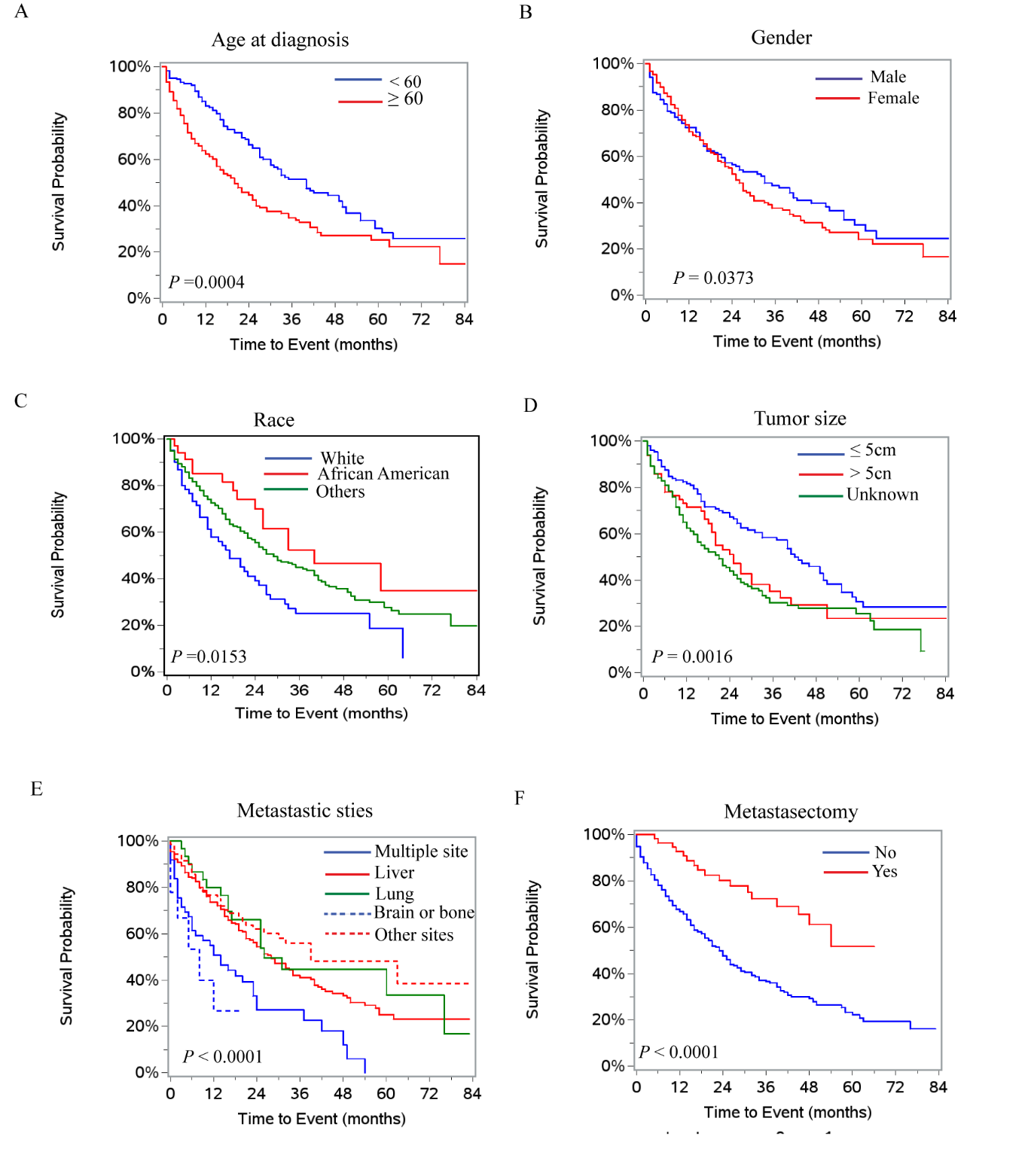


Fig. S3. Kaplan-Meier survival curves for patients with stage T1 colorectal cancer. Upper panel: Association between histology and cancer specific survival in (A) all patients. (B) Patients without distant metastases. Lower panel: Association between histology and overall survival in (C) all patients. (D) Patients without distant metastases.


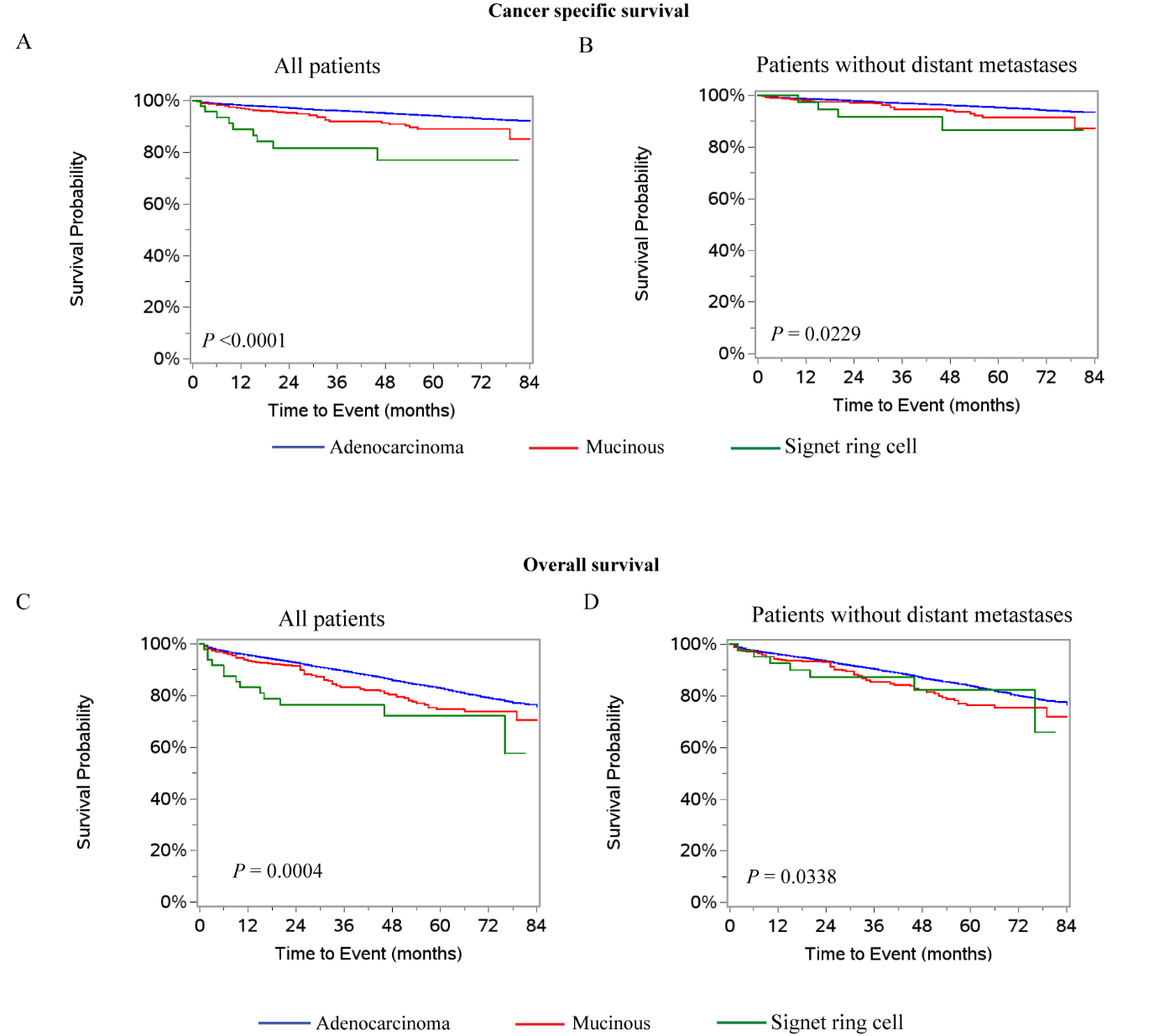

Supplement: Supplementary file 1 — Supplementary Information. [file 41598_2021_87929_MOESM1_ESM.docx]
